# Meteorological Journal

**Published:** 1823-01

**Authors:** 


					METEOROLOGICAL JOURNAL.
By Messrs. William Harris and Co. 50, Holborn, London.
From November 20, 1822, to December 19, 1822.
Rain
gaug,
therm.
Nov
20 .37152
!1 (f .05850
22 .12
23
24 .05
25 .11
26 .13
27 .03
28 O -14
29 .07
30 .10
Dcc
1 .15
2 .45
3
4 .66
5
6
7
8
9
10
11
12
13
14
15
16
17 .07
18
19
?JO 32
29.50
29.41
29.70
29.42
29.71
29.42
29.49
29.45;
29.33
29.31
29.20
29.18
29.91
29.10
29.46
29.60
29.30
29.78
30.06
29.97
30.18
30.38
30.38
30.20
30 10
29.90
29.96
30.16
30.0-1
30.06
29.47
29-64
29.48
29.4-8
29.50
29.40
29.32
29.53
29.10
29.09
29.29
28.90
28.91
29.58
29.23
29.23
29.56
29.92
50.00
29.98
30.50
30.59
50.52
50.15
50.02
29.90
50.12
30.09
50.04
50.15
' DE
LUC'S
IIYG.
ATMOSPHERIC
VARIATION.
9 A.M.
ssw
WSW
wsw
vvsw
sw
wsw
wsw
w
ssw
W var.
W
wsw
w
NE
w
w
NW
WNW
WSW
wsw
NNE
NE
ESE
E
72 SE
71 SE
71 NE
W
78 NE
72 E
10p.m.
SW
jWSW
SW
w
SSW
SW
wsw
w
SW
WNW
NW
WSW
ssw
N
w
SW
NW
WNW
WSW
ws w
NE
EN7E
ESE
SSE
SE
E
N
WNW
ENE
E
9 A.M.
Rain
Showery
Fine
Fine
Fine
Rain
Rain
Showery
Rain
Fine
Fine
Cloudy
Fine
Cloudy
Fog
Fine
Fine i
Fog
Fine
Fine
Fine
Fog
Fine
Fine
Fine
Fine
Fine
Fine
Cloudy
Fine
2 P.M.
Rain
Fine
Rain
Cloud
Rain
Sho'iy
('loud.
Fine
Cloud.
Rain
Rain
Rain
Cloud.
Fine
Rain
Cloud.
Fine
Fine *
Fine
Rain
Fine
Fog
Fine
Fine
Fok
I he quantity of rain that lias fallen in the mouth of November,
was 3 inches and 15.1o0ths.
no. 2b7-

				

